# Case Report: Spontaneous rupture of an internal thoracic artery aneurysm: a rare life-threatening emergency and Its therapeutic challenges

**DOI:** 10.3389/fsurg.2026.1777076

**Published:** 2026-04-29

**Authors:** Xun Guo, Zhuohang Liu, Zheng Liu, Hongquan Fan

**Affiliations:** 1 Comprehensive Surgical Nursing Platform, The First Hospital of Jilin University, Changchun, China; 2 Radiology Department, The First Hospital of Jilin University, Changchun, China; 3 Emergency Surgery Department, The First Hospital of Jilin University, Changchun, China; 4 Emergency Nursing Platform, The First Hospital of Jilin University, Changchun, China

**Keywords:** diagnosis and treatment, emergency care, internal thoracic artery (ITA), spontaneous rupture, therapeutic challenges

## Abstract

Internal thoracic artery (ITA) aneurysms are exceedingly rare vascular anomalies that often remain asymptomatic until rupture. Rupture can precipitate acute hemothorax, hemorrhagic shock, and death if not rapidly diagnosed and managed. We report the case of a 42-year-old previously healthy female who presented to the emergency department with sudden-onset chest, abdominal, and back pain. Initial CT imaging elsewhere revealed pleural effusion, a non-specific finding. Upon transfer to our institution, emergency computed tomography angiography (CTA) demonstrated a ruptured left ITA pseudoaneurysm with active contrast extravasation and a large left hemothorax. Emergent transcatheter arterial embolization using microcoils achieved definitive hemostasis. Ultrasound-guided chest tube drainage was subsequently performed. The patient recovered uneventfully and was discharged without complications. This case highlights a critical diagnostic pitfall in emergency medicine—ruptured ITA aneurysm masquerading as simple pleural effusion—and underscores the pivotal role of CTA and endovascular therapy in optimizing outcomes. We also review the literature on etiology, diagnostic challenges, and evolving therapeutic strategies for this rare but fatal condition.

## Introduction

Internal thoracic artery (ITA) aneurysms, also historically termed internal mammary artery (IMA) aneurysms, represent a rare vascular entity typically remaining asymptomatic prior to rupture (1). Rupture constitutes a life-threatening emergency, manifesting as acute chest pain, dyspnea, and hemothorax, which can rapidly progress to hemorrhagic shock. Due to the non-specific nature of presenting symptoms, prompt diagnosis is challenging and requires a high index of suspicion. Computed tomography angiography (CTA) is the imaging modality of choice, enabling definitive diagnosis by demonstrating the aneurysm, active extravasation, and associated hemothorax (2). Once diagnosed, immediate intervention is critical. While traditional management involved thoracotomy with aneurysm resection or vessel ligation, advances in endovascular techniques have established transcatheter arterial embolization (TAE) as a safe, minimally invasive, and highly effective first-line treatment, particularly in hemodynamically unstable patients (3, 4). While ITA aneurysms are consistently described as exceedingly rare in the literature, precise incidence data are unavailable. To date, fewer than 50 cases have been reported worldwide, predominantly as single case reports or small case series. The true incidence is likely underestimated, as many remain asymptomatic and undetected. The rarity of this condition contributes to the diagnostic challenges faced by emergency physicians.We report a case of spontaneous ruptured ITA aneurysm successfully managed with emergency endovascular embolization, emphasizing diagnostic strategies and therapeutic considerations relevant to emergency physicians and interventionalists.

## Case presentation

A 42-year-old woman with no significant past medical history presented to our emergency department with acute-onset chest, abdominal, and back pain that began one day prior, without identifiable precipitating factors. At a local hospital, a non-contrast chest CT revealed a left pleural effusion. Despite receiving analgesics and intravenous fluids, her symptoms persisted, prompting transfer to our tertiary care center.

On arrival, she was tachycardic (heart rate 115 bpm) and hypotensive (blood pressure 88/52 mmHg). Her respiratory rate was 22 breaths/min, and oxygen saturation was 94% on room air. Laboratory studies revealed a hemoglobin of 117 g/L, lactate of 3.8 mmol/L, and a normal coagulation profile. An emergency electrocardiogram showed no ischemic changes.

**Figure 1:**
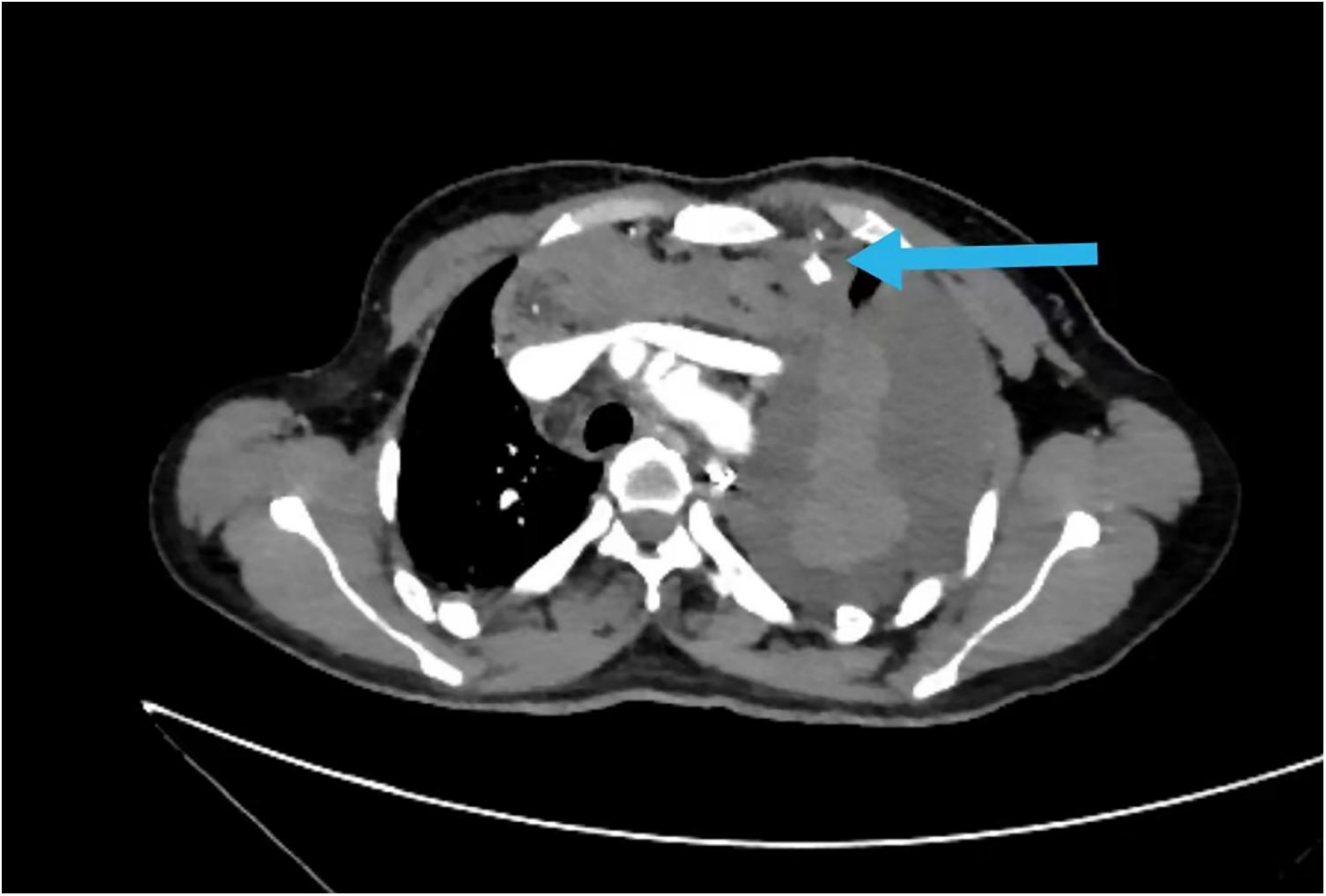
Rupture of IMA aneurysms (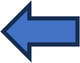) in CTA scan of the thoracic and abdominal vessels.

**Figure 2:**
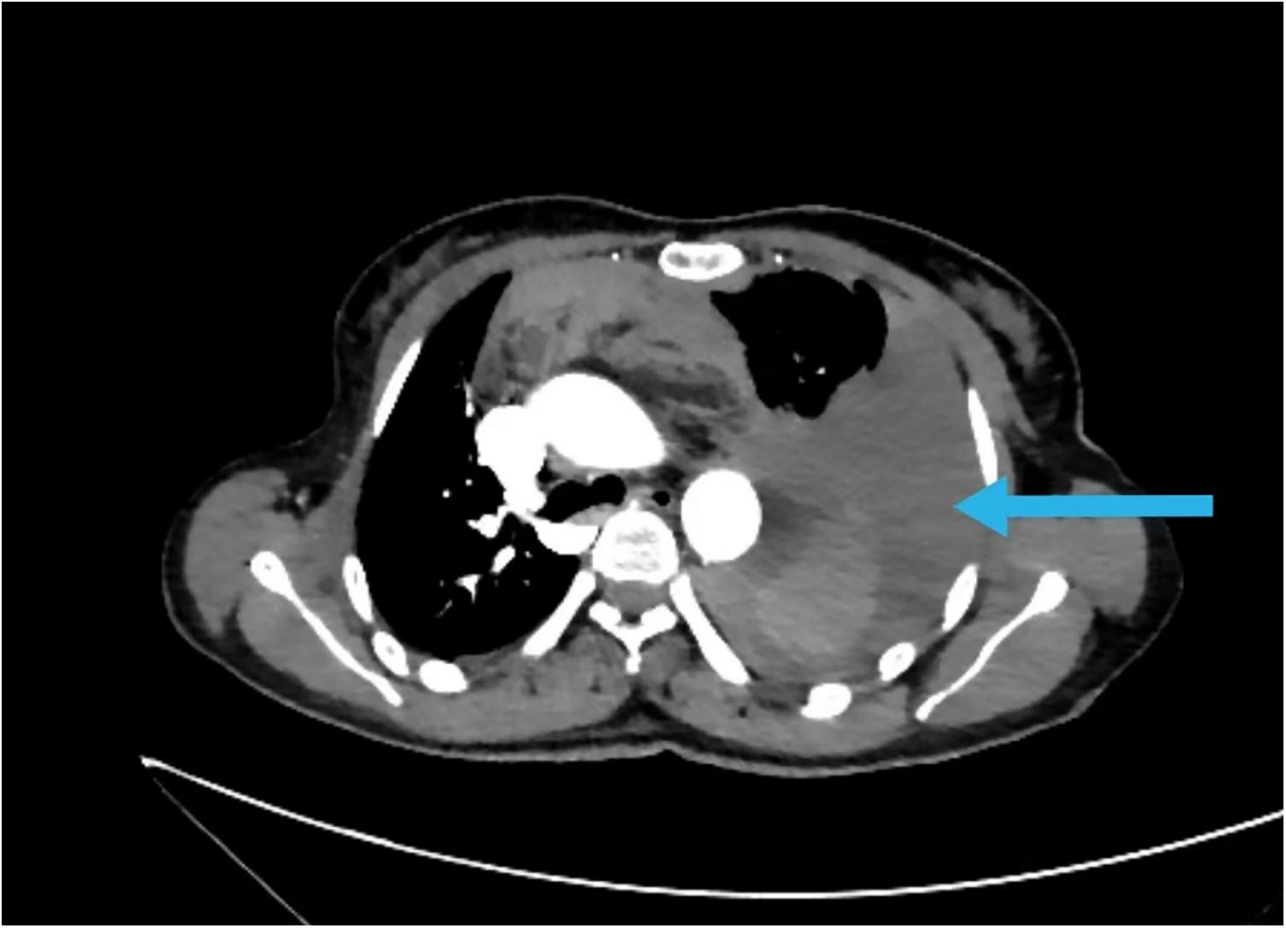
Hematoma and effusion in the mediastinum and left thoracic cavity (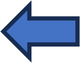).

Given the unexplained hemothorax and hemodynamic instability, an urgent CTA of the chest and abdomen was performed. This revealed a small (0.7 × 1.2 cm) saccular pseudoaneurysm arising from the proximal-to-mid segment of the left internal thoracic artery, just posterior to the sternum (Figure 1). Active extravasation of contrast was evident, along with a large left hemothorax and associated mediastinal hematoma (Figure 2). These findings confirmed the diagnosis of a ruptured ITA pseudoaneurysm with active hemorrhage.

**Figure 3:**
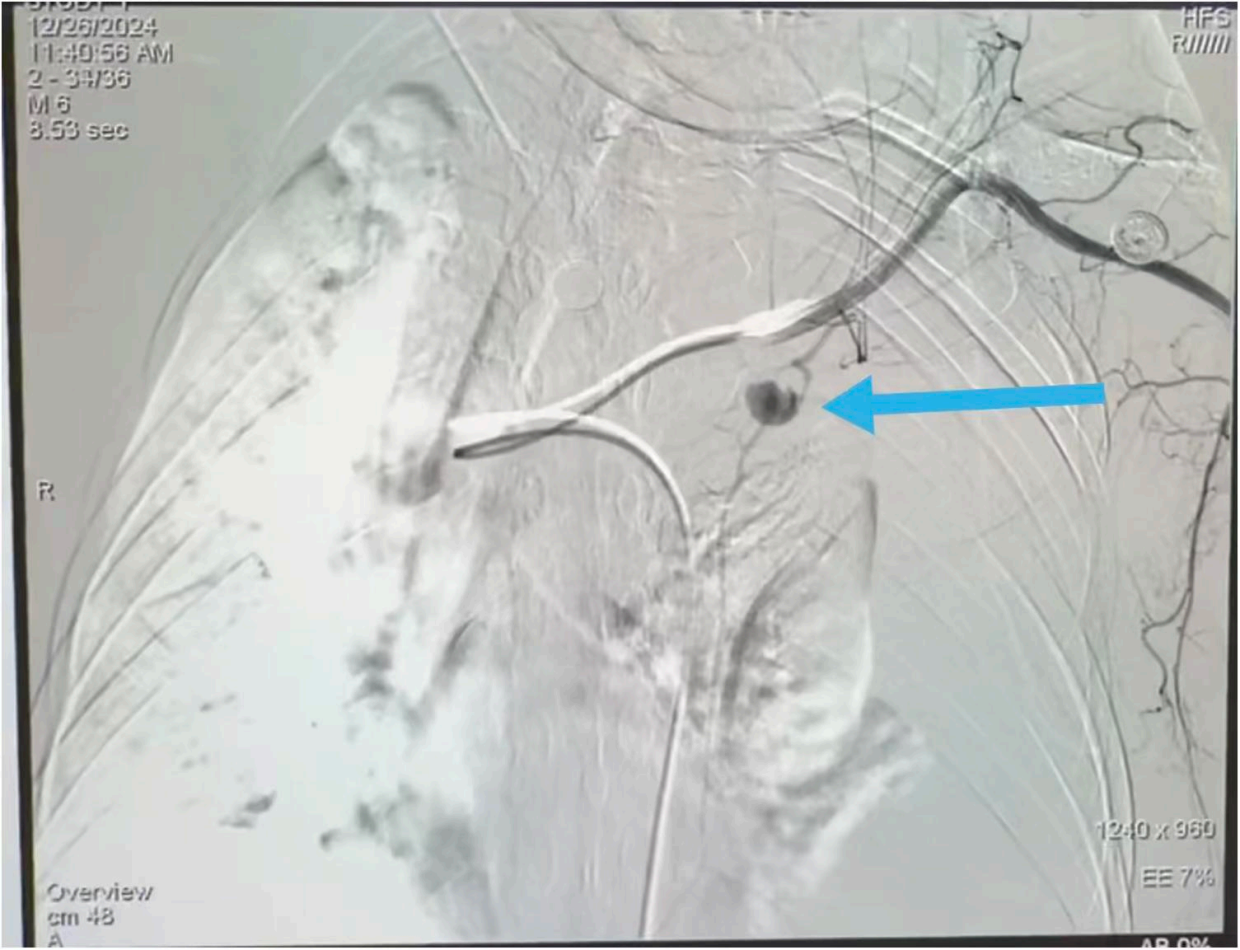
Rupture of IMA aneurysms (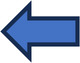) under contrast injection revealed rapid extravasation from the aneurysm into the pleural cavity.

The patient was emergently transferred to the interventional radiology suite. Through a right common femoral artery approach, selective left internal thoracic arteriography was performed, which confirmed the pseudoaneurysm and demonstrated rapid contrast extravasation into the left pleural cavity (Figure 3). Through a 5-Fr right common femoral artery sheath, a 5-Fr internal mammary catheter was used to selectively cannulate the left subclavian artery. A 2.4-Fr microcatheter was then advanced coaxially into the left internal thoracic artery. Angiography confirmed the pseudoaneurysm and active extravasation. Three 4 mm × 10 cm and two 3 mm × 6 cm detachable microcoils were deployed sequentially in the parent artery proximal and distal to the aneurysm neck. Post-embolization angiography demonstrated complete occlusion of the ITA with no residual contrast extravasation, confirming technical success. The parent artery was successfully embolized using multiple microcoils placed proximally and distally to the aneurysm neck, achieving complete cessation of flow and extravasation on post-embolization angiography.

Following the procedure, an ultrasound-guided 28-French chest tube was inserted into the left pleural space, draining 1,200 mL of dark bloody fluid immediately, with continued output of 300 mL over the next 24 h. The patient was admitted to the intensive care unit for monitoring. She received transfusion of two units of packed red blood cells (hemoglobin post-transfusion 99 g/L), supplemental oxygen, and broad-spectrum antibiotics (cefazolin) for 48 h. Her hemodynamic status rapidly stabilized, and vasopressors were not required. The chest tube was removed on postoperative day 5 after output ceased and lung expansion was confirmed. A follow-up CT on postoperative day 11 demonstrated near-complete resolution of the hemothorax and no evidence of recurrent bleeding. The patient was discharged home on postoperative day 14 in good condition. At 6-month follow-up, she remained asymptomatic with no recurrence.

## Discussion

Spontaneous rupture of an ITA aneurysm is a rare but catastrophic event that must be considered in the differential diagnosis of acute hemothorax, particularly when accompanied by hemodynamic instability (5), This case underscores several key learning points for emergency physicians and highlights the evolving role of endovascular therapy.

## Etiology and clinical presentation

The etiologies of ITA aneurysms are diverse and include atherosclerosis, trauma (blunt or penetrating), iatrogenic injury (e.g., post-sternotomy, central venous catheter placement, or coronary artery bypass grafting), infection (mycotic aneurysm), and vasculitides such as Takayasu arteritis or connective tissue disorders (e.g., Marfan syndrome, Ehlers-Danlos syndrome) (6–10). However, as in the present case, a significant proportion remain idiopathic, with no identifiable risk factors (11).

Prior to rupture, these aneurysms are typically asymptomatic. When symptoms do occur, they may include non-specific chest discomfort, cough, or dyspnea. Rupture classically presents with sudden, severe chest or back pain, often followed by dyspnea, signs of hypovolemia, and rapid progression to shock if not emergently treated (12, 13). The presence of a hemodynamically significant hemothorax in a young patient without trauma should prompt consideration of a vascular etiology.

## Patient perspective, genetic testing, and screening for concomitant aneurysms

The sudden, life-threatening rupture of a previously unknown aneurysm is a profoundly unsettling experience for patients. In our case, the patient expressed significant anxiety about the possibility of recurrence or the presence of other undetected aneurysms, which prompted her request for follow-up CTA at 6 months. This psychological impact should not be underestimated and warrants attention in post-discharge care, including appropriate reassurance and counseling.

Genetic testing: The role of genetic testing in patients with apparently spontaneous ITA aneurysm rupture is not clearly defined. In the absence of clinical features suggesting a specific heritable connective tissue disorder (e.g., Marfan syndrome, Ehlers-Danlos syndrome, Loeys-Dietz syndrome, vascular Ehlers-Danlos syndrome), routine genetic testing is not currently recommended (14, 15). However, a detailed family history should be obtained, and if features suggestive of an inherited condition are present (e.g., family history of aortic dissection or sudden death, skeletal features, lens dislocation, skin hyperextensibility), referral to clinical genetics for evaluation and possible targeted genetic testing is appropriate. In our patient, no such features were identified, and genetic testing was not pursued.

Screening for concomitant aneurysms: The literature on coincidence of ITA aneurysms with aneurysms at other locations is limited to case reports. Some cases have described ITA aneurysms occurring in patients with aortic aneurysms, particularly in the setting of connective tissue disorders or vasculitides (5, 9, 13). However, in patients with apparent idiopathic, isolated ITA aneurysms, the risk of concomitant aneurysms elsewhere is unknown. Given the lack of evidence, routine whole-body imaging cannot be mandated. A pragmatic approach involves: (1) thorough review of the initial CTA (which typically includes the chest and upper abdomen) for other vascular abnormalities; (2) baseline imaging of the abdominal aorta if not adequately visualized on the initial study; and (3) clinical vigilance for symptoms referable to other vascular territories during follow-up. Shared decision-making should guide any additional screening, taking into account patient anxiety and preferences.

Future research should focus on establishing registries for rare vascular aneurysms to better understand their natural history, genetic underpinnings, and association with multifocal disease.

## Diagnostic approach: the critical role of CTA

Our case illustrates a common diagnostic pitfall: an initial non-contrast CT demonstrating only pleural effusion, a finding easily attributed to more common causes such as infection, malignancy, or pulmonary embolism. In the setting of hemodynamic instability or unexplained hemothorax, contrast-enhanced imaging is mandatory. CTA offers several advantages: it is rapid, non-invasive, and provides detailed anatomical information, including the precise location and morphology of the aneurysm, the presence of active extravasation (a “contrast blush”), and the extent of hemothorax and hematoma (2, 16). This information is crucial not only for diagnosis but also for planning intervention. In our patient, CTA definitively identified the ruptured ITA pseudoaneurysm as the bleeding source, transforming a vague diagnosis of “pleural effusion” into a precise surgical target. An important anatomical consideration is that the internal thoracic artery courses posterior to the costal cartilages and anterior to the parietal pleura, within the endothoracic fascia. Rupture of an ITA aneurysm initially causes extrapleural hematoma formation, separating the parietal pleura from the chest wall. Only when the pleura is secondarily breached does blood enter the true pleural cavity, resulting in the hemothorax observed on imaging. This pathophysiological sequence explains why CTA may demonstrate both mediastinal/extrapleural hematoma and pleural effusion.

## Therapeutic evolution: from open surgery to endovascular intervention

Historically, management of ruptured ITA aneurysms required open surgical approaches, including thoracotomy or sternotomy with aneurysm resection, ligation of the parent vessel, or vascular reconstruction (13, 17). While effective, these procedures are associated with significant morbidity, particularly in the setting of active hemorrhage and hemodynamic compromise. The risks of general anesthesia, prolonged operative time, and surgical trauma are considerable.

The advent of endovascular techniques has revolutionized the management of these lesions. Transcatheter arterial embolization (TAE) offers a minimally invasive alternative that can be performed rapidly, often under local anesthesia and conscious sedation (18). As demonstrated in our case, super-selective catheterization of the ITA allows precise placement of embolic agents (coils, plugs, or liquid embolics) to occlude the aneurysm and its parent vessel, achieving immediate hemostasis. Multiple case series have confirmed the high technical and clinical success rates of TAE for both iatrogenic and spontaneous ITA injuries, with low complication rates (19–21). For hemodynamically unstable patients, TAE is particularly advantageous as it avoids the added physiological insult of major surgery and allows for rapid control of bleeding. The choice of surgical access—sternotomy, thoracotomy, or video-assisted thoracoscopic surgery (VATS)—should be reserved for cases where endovascular therapy fails, is unavailable, or when concurrent procedures (e.g., evacuation of a large clotted hemothorax) are necessary. VATS represents a less invasive surgical option but may be challenging in the setting of active, massive hemorrhage.

## Management of incidentally discovered non-ruptured ITA aneurysms

The management of incidentally discovered, non-ruptured ITA aneurysms is not standardized due to the extreme rarity of this condition and the absence of prospective studies or consensus guidelines. Treatment decisions must therefore be individualized based on several factors: aneurysm size and morphology, rate of growth if serial imaging is available, etiology(e.g., connective tissue disease, vasculitis), patient age and comorbidities, and the presence of symptoms.

Some authors advocate for prophylactic intervention in all cases given the potentially catastrophic consequences of rupture (12, 18). Endovascular embolization, being minimally invasive with low morbidity, is an attractive option for asymptomatic aneurysms. Others suggest that small, stable, incidentally detected aneurysms in patients without risk factors for rupture may be managed conservatively with serial imaging surveillance (11, 16). However, the natural history of untreated ITA aneurysms remains unknown, and no evidence-based size threshold for intervention has been established.

In patients with underlying connective tissue disorders (e.g., Marfan syndrome, Ehlers-Danlos syndrome) or vasculitides (e.g., Takayasu arteritis), a lower threshold for intervention may be warranted due to the risk of progressive arterial wall weakening and multifocal disease. Until more data become available, management should be determined through multidisciplinary discussion involving vascular surgery, interventional radiology, and cardiology, with shared decision-making that incorporates patient preferences.

## Post-intervention management

Comprehensive post-procedural care is essential. Chest tube drainage is critical to evacuate the hemothorax, prevent fibrothorax and empyema, and allow lung re-expansion. Ongoing resuscitation with blood products as needed, respiratory support, and antibiotic prophylaxis are standard adjuncts. Close monitoring for re-bleeding is necessary, though recurrence after successful embolization is rare. Follow-up imaging is advisable to confirm hematoma resolution and exclude the development of new aneurysms, particularly in idiopathic cases.

We have summarized the information from previously reviewed published articles on ITA aneurysms, including author/year, etiology/associated condition, clinical presentation (ruptured/unruptured), treatment, and key differences from our case (Table 1).

**Table 1:** Summary of reported internal thoracic artery aneurysm cases and comparison with the present case.

No.	First Author/Year	Etiology/Associated Condition	Rupture	Treatment	Key Differences from Our Case
1	Present case (2026)	Idiopathic	Yes	Coil embolization	–
2	Lawani et al. 2021 (5)	Coronary artery bypass graft	No	Covered stent	Incidental finding; iatrogenic etiology; different intervention
3	Ahmed et al. 2024 (12)	Idiopathic	No	VATS resection	Elective surgery; no active bleeding
4	Siegel et al. 2021 (13)	Marfan syndrome	Yes	Endovascular? (details limited)	Underlying connective tissue disorder; possible multifocal aneurysms
5	Miyazaki et al. 2019 (18)	Idiopathic	No	Thoracoscopic resection	Elective surgery; no rupture
6	Sueyoshi et al. 2020 (19)	Takayasu arteritis	No	Stent-graft	Inflammatory etiology; unruptured
7	Deng et al. 2024 (20)	Idiopathic	No	Coil embolization	Asymptomatic; elective embolization
8	Franciosi et al. 2024 (21)	Traumatic(Cesarean section)	Yes	Coil embolization	Trauma-related; identical endovascular approach
9	Kwan et al. 2024 (16)	Idiopathic	No	Conservative	Small, asymptomatic; no intervention
10	Müdüroğlu et al. 2019 (11)	Idiopathic?(details limited)	Unknown	Surgical ligation	Unclear rupture status; open surgery
11	Toscano et al. 2019 (6)	Iatrogenic	Unknown	Embolization	Iatrogenic cause; elective
12	Inoue et al. 2021 (7)	Post-sternotomy	No	Embolization	Post-surgical; unruptured
13	Ho et al. 2018 (8)	IgG4-related disease	No	Hybrid (stent + resection)	Inflammatory; multifocal
14	Koutouzis et al. 2007 (17)	Spontaneous dissection (ITA graft)	No	Conservative	Graft dissection, not aneurysm

This table includes representative cases from the literature cited in our manuscript. For cases where rupture status was not explicitly stated, it is inferred from the available information.

VATS, video-assisted thoracoscopic surgery; ITA, internal thoracic artery.

## Limitations

As a single-case report, our findings have inherent limitations in generalizability. The long-term durability of coil embolization for ITA aneurysms requires further study, and life-long surveillance may be considered, although our patient showed no recurrence at short-term follow-up.

## Conclusion

Spontaneous rupture of an ITA aneurysm is a rare but devastating cause of acute hemothorax and hemorrhagic shock. Emergency physicians must maintain a high index of suspicion for this entity in patients presenting with unexplained chest pain and pleural effusion, particularly when accompanied by hemodynamic compromise. CTA is the cornerstone of rapid and accurate diagnosis. Emergency endovascular embolization has emerged as the preferred first-line treatment, offering a safe, minimally invasive, and highly effective means of achieving hemostasis, even in unstable patients. This case reinforces the critical importance of a multidisciplinary approach—integrating emergency medicine, radiology, and interventional vascular surgery—in optimizing outcomes for these challenging vascular emergencies.

## Data Availability

The raw data supporting the conclusions of this article will be made available by the authors, without undue reservation.
